# Principles of artificial intelligence and its application in cardiovascular medicine

**DOI:** 10.1002/clc.24148

**Published:** 2023-09-18

**Authors:** Heinrich Wieneke, Ingo Voigt

**Affiliations:** ^1^ Department of Cardiology and Angiology, Contilia Heart and Vascular Center Elisabeth‐Krankenhaus Essen Essen Germany

**Keywords:** artificial intelligence, cardiology, cardiovascular medicine, CNN, deep neural network, machine learning

## Abstract

Artificial intelligence (AI) represents a rapidly developing field. Its use can improve diagnosis and therapy in many areas of medicine. Despite this enormous progress, many physicians perceive it as a black box and are skeptical about it. This review will present the basics of machine learning. Different classifications of artificial intelligence, such as supervised versus unsupervised and discriminative versus generative AI, are given. Analogies to human intelligence are discussed as far as algorithms are oriented toward it. In the second step, the most common models like random forest, k‐means clustering, convolutional neural network, and transformers will be presented in a way that the underlying idea can be understood. Corresponding medical applications in cardiovascular medicine will be named for all models, respectively. The overview is intended to show that the term artificial intelligence covers a wide range of different concepts. It should help physicians understand the principles of AI to make up one's minds about its application in cardiology. It should also enable them to evaluate results obtained with AI's help critically.

## Graphical abstract

Artificial intelligence (AI) represents a rapidly developing field. This review will present the basics of AI. Different classifications of AI, like supervised versus unsupervised and discriminative versus generative AI, are given. Analogies to human intelligence are discussed as far as algorithms are oriented toward it. The second step will present the most common models like random forest, k‐means clustering, convolutional neural networks, and transformers to understand the underlying ideas. Corresponding medical applications in cardiovascular medicine will be named for all models.

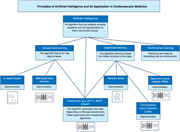

## 1 INTRODUCTION

In recent years, there has been hype regarding artificial intelligence (AI) in medicine. Although there are various definitions of human intelligence, it can roughly be described as the ability to analyze complex situations and act appropriately on them. In analogy, AI can be seen as a method to achieve the same behavior and goals by artificial means, predominantly computer algorithms. The term “artificial intelligence” was initially coined by John McCarthy, who referred to it as “a system which is to evolve intelligence of human order.”^1^

The significant advances in AI are accompanied by mystification, especially in the lay press. This has led to terms like “black box AI” since humans do not intuitively understand results. However, there are many technical achievements in the modern world that humans need help understanding at first glance. Many AI models can be traced back to simple mathematical models. Only the quantity of operations and data makes it impossible to trace results in detail. On the other hand, this is also the main reason for the continued growth of AI applications. The availability of large computing capacities, big data, and mathematical algorithms allows us to recognize patterns in a way that was impossible before.

This paper aims to explain the basics of AI. In this context, it will focus on machine learning (ML). ML is a subcategory of AI. ML is the capability to make predictions and decisions based on data. AI, on the other hand, is an umbrella term that includes further subcategories like natural language processing (NLP), computer vision (CV), text‐to‐speech models, and robotics, in addition to ML. Especially a new field of AI called foundation models, has recently increased strongly in importance. The term foundation model, coined at Stanford in 2021, describes AI systems that have been trained with a large amount of data so that they can attend to different tasks. The models can then be adapted for specific tasks (fine‐tuning). The most well‐known foundation models are the large language models (LLMs) GPT‐n (OpenAI), BERT (Google), LLaMA (Meta AI), and Claude (Anthropic AI). LLMs embrace transformer architectures, which are mentioned at the end of the review.

Selected models of ML and their applications based on these principles will be presented in this review. Supporting Information: Table S1 gives an overview of the most important algorithms, examples of their application in cardiology, and the specific problem they are designed to solve.

It will also be shown that many concepts have been adopted from brain research. Since many algorithms have only been developed recently, their medical application is often still at the experimental or evaluation stage. Many applications have yet to find their way into routine clinical use.

## 2 GENERAL PRINCIPLES OF AI

### 2.1 Nonlearning versus learning systems

A categorization of AI into learning and nonlearning algorithms is introduced to facilitate further understanding. In nonlearning systems, algorithms react in a predefined way in particular situations. The system always responds in the same way. It, therefore, does not learn. Classic chess programs, for example, are programmed this way.

In contrast, more elaborated AI systems use an approach where the systems learn. In classical computing, an algorithm, usually called a program, is fed with input data, which are processed by the program, and consequently, the output is given back to the user. The simplest example in this context is a pocket calculator. While executing a mathematical operation, the actual program of the calculator is not changed, which means that the program is not learning. This is different from learning AI algorithms. When these algorithms are fed with data, the algorithms change while processing, which means that the algorithms learn. The name “ML” refers to this characteristic. Principally learning systems can be divided into neural networks that mimic the human brain (deep learning) and alternative architectures.

#### 2.1.1 Learning systems mimicking the human brain

**Figure 1:**
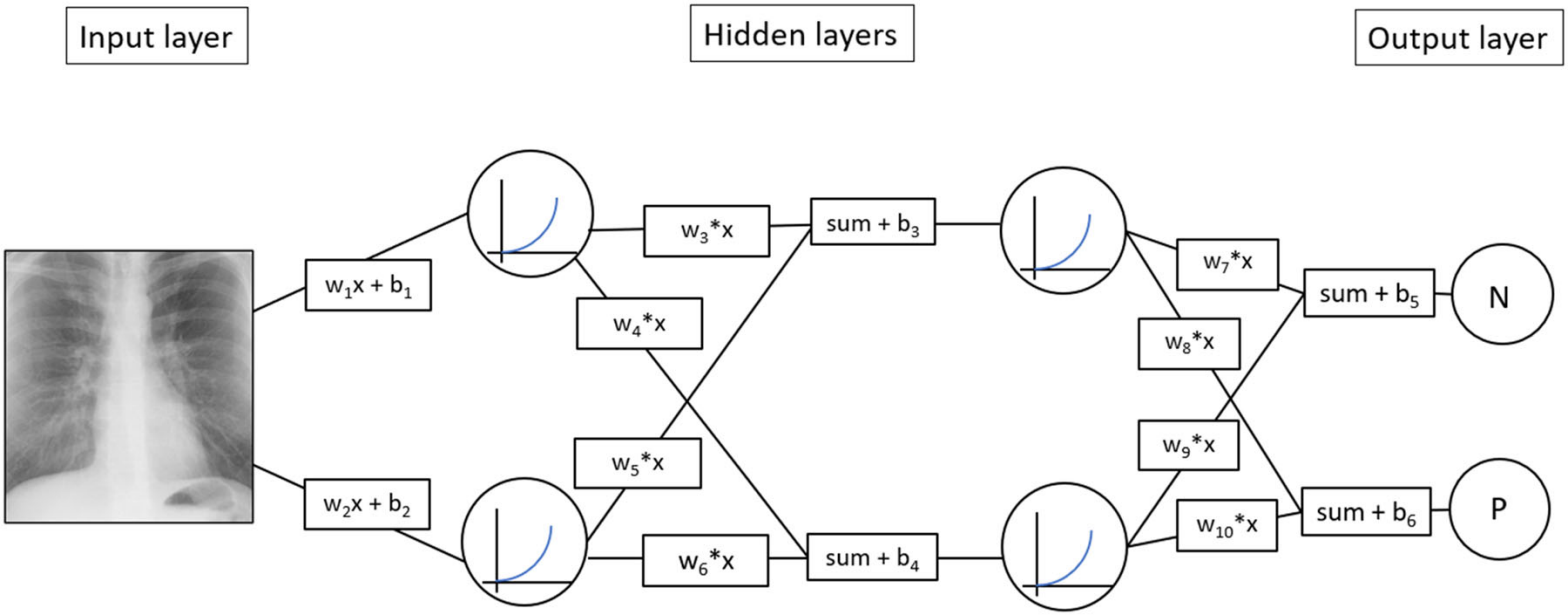
The basic architecture of deep neural networks (DNN). The figure shows a very basic DNN. In this example, the purpose of the network is to assign images to two classifications, N and P. The pixel values of a picture, in the present case a chest X‐ray, are fed into this network. The input values are multiplied by weights (*w*_x_), which are random in the first step. To these values, a bias is added (*b*_x_). The sum of the weights and the biases represent the *x*‐value of the activation function. Specific activation functions fitting particular problems can be used. The output of the activation function, the *y*‐values, is the input of the next layer. These basic steps are repeated for any node of the network. The network's final output is a probability of whether the picture belongs to the N or P category. This output probability is compared to the actual value, and a loss is calculated. The following steps aim to optimize weights and biases to minimize the loss or, in other words, minimize the difference between the prediction and the ground truth. This mathematical operation is called backpropagation.

Deep learning is a subclass of ML using artificial neural networks (ANN) inspired by the idea of replicating brain architecture. The natural brain is composed of neurons that are arranged in layers. These neurons get inputs through dendrites. When the summed inputs reach an activation potential, neurons generate an action potential forwarded through the axon to other neurons in other layers. In analogy to the neuron, the most basal component of an ANN is called a node. This node gets input from other nodes. These inputs are called weights and biases that are adjusted during learning. A particular node's inputs are summed and fed to a nonlinear activation function, which calculates an output given to the next layer. In analogy to the biological brain, ANNs can be composed of many layers, so they carry the adjective deep. The first layer is also known as the input layer, and the last is called the output layer. The layers in‐between are the hidden layers (Figure 1). This architecture is the backbone of every ANN. Frank Rosenblatt programmed the first ANN in the 50 s of the last century.^2^ This basic form is called a multilayer perceptron or vanilla neural network.

But how does the ANN learn? Let us illustrate this with the example of supervised learning. The algorithm's task is to classify images, for example, chest X‐rays showing lung cancer or not. The process can be divided into two steps: In the first step, the ANN is trained with labeled data. To explain it simply, the input data are the pixels of the picture, and the weights are a measure of the magnitude each pixel contributes to classification. Many data instances propagate through the network. Propagation in this context means that the algorithm tries to identify features, pixels in the case of pictures, that are important for classification. The output of each data instance is compared with the corresponding label, and an error rate is calculated. This error rate, also called loss, is the difference between the predicted value of the network and the correct label, also called ground truth. This loss is then given back to the algorithm for adjusting the weights. This process is called backpropagation. The learning process aims to minimize the difference between the prediction of the network and the ground truth. Thus, the algorithm is changed during these repeated data propagations and backpropagations. In a mathematical sense, learning involves adjusting the weights and biases. After the learning phase, the trained network can classify unknown data. To stay with the chest X‐ray example, in the second step, unknown and unlabeled data are fed to the ANN, and the ANN gives a probability of whether lung cancer is present (Figure 1).

After establishing an ANN, checking whether an AI system performs sufficiently in the real world is essential. For this purpose, it is evaluated how correctly the algorithm classifies a test data set. In this context, an evaluation matrix comprising, among others, accuracy, F1‐score, sensitivity, and C‐statistic can be used.

These concepts are not new, but the accumulation of big data and the computational capability to apply the corresponding algorithms put scientists in the position to recognize patterns in data that are not intuitive to the human brain.

### 2.2 Supervised versus unsupervised learning

Although learning is not a component of all forms of AI, it can nevertheless be described as the most crucial technique for accomplishing specific tasks. This subfield of AI is called ML. When ML uses the architecture of a neural network, this is also referred to as deep learning. Thus, deep learning is a subcategory of ML.

In this context, two kinds of learning are distinguished: supervised and unsupervised. As explained above, in supervised learning, the algorithm is first trained with clearly classified data, for example, pictures of lung cancer or not. The algorithm extracts features from these data. In the second step, the algorithm classifies data based on these features.

In contrast, the principal concept of unsupervised learning is detecting unknown patterns in unlabeled data without human help. With this concept, there is no distinct learning with labeled data. The algorithm analyzes the data and detects patterns by itself. This clustering uses specific mathematical operations like calculating the Euclidian distance for different center points.

Using this approach, subtypes in a group of patients with an umbrella diagnosis can be identified. For example, five subtypes of heart failure could be identified in two population‐based datasets in the United Kingdom.^3^

### 2.3 Discriminative versus generative AI

Discriminative algorithms divide a data set into groups that share common properties. Labels are assigned to these groups in the learning process. For example, an algorithm can learn to “discriminate” normal X‐rays images from images with malignancy. To achieve this goal, neural or conventional architecture algorithms can be used.

The generative algorithms are distinguished from the discriminative ones. Generative AI can generate new and original content like pictures and text using patterns recognized in existing data. These algorithms can be used for data augmentation as in many fields only a limited amount of training data are available.^4^

## 3 SPECIAL AI ALGORITHMS AND THEIR APPLICATIONS

### 3.1 Random forest

#### 3.1.1 Intuition

A typical example of ML is random forest.^5^ Unlike the algorithms presented below, it is not based on a neural network architecture but can still learn. It is a supervised learning algorithm built on an ensemble of decision trees, which gives rise to its name. It is one of the most often used ML algorithms for classification and correlation tasks.

**Figure 2:**
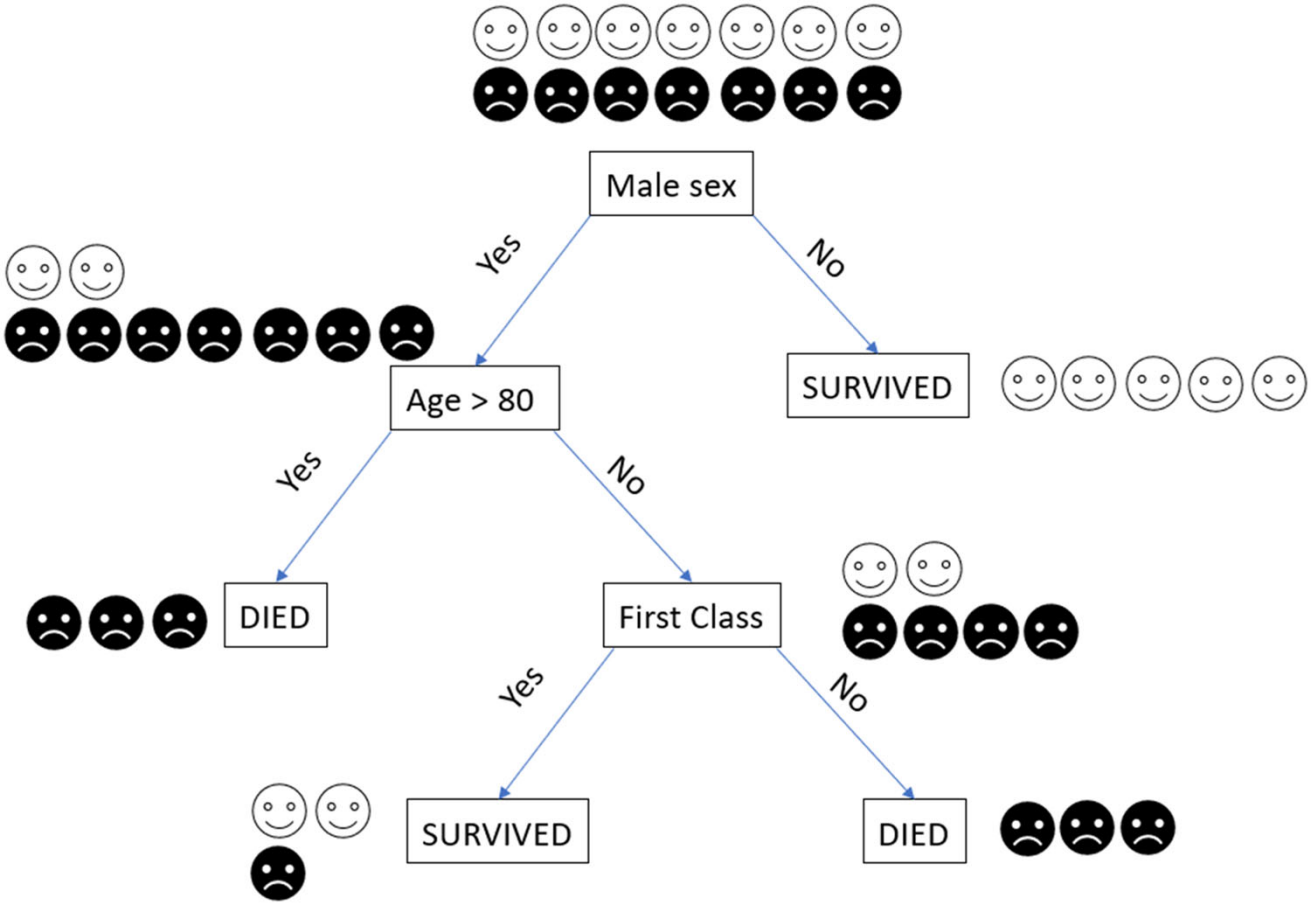
Random forest. This figure shows a decision tree, the building block of the random forest algorithm. We take a small sample (14 passengers, 7 survivors, and 7 nonsurvivors) of the titanic data set.^6^ An algorithm will be created that predicts whether a passenger will survive based on the available data. There are two classes (leave nodes): survivor 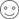 nonsurvivor 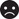. There are three decision nodes: sex, age, and class. The first decision node, sex, leads at the right to a pure node, which means that there are five females who all survived. Thus, no further splitting is necessary. On the left are nine male passengers; two survived, and seven died. Thus, further splitting is mandatory. In this arm, the next decision node is age >80 years. There are three male passengers older than 80 years who all died. It is a leave node with no further splitting. There were six male passengers ≤80 years, two survived, and four died. Another split concerning class membership is done. As you see, there are three male passengers ≤80 years in the first class, from which two survived, and one died. The leave node, in this case, is not pure. Thus, this decision tree is not perfect. In a second step, now new passengers can be classified based on this decision tree. When the new passenger runs into the nonpure leave, majority voting is done.

The building block of the random forest is the decision tree. A decision tree consists of decision nodes and leave nodes. The decision nodes are the available parameters or attributes of the data set. The leave nodes are the classes. The decision nodes split data according to a statement about these parameters. The statement can be true or false, and the way could go down to the next decision node or a leaf node. In each data set, there are many possible splitting conditions. The ML part in this context is to find the best splitting parameters with the best cut‐off values to classify the values. The data set values are taken for an individual decision tree, and different configurations of decision nodes and cut‐off values are evaluated for optimal separation (Figure 2). A disadvantage of this algorithm is that decision trees are too adapted to the training data set, called overfitting. That makes them prone to generalization errors. This is where the random forest algorithm comes into play.

The principal idea of random forest is to create many of these decision trees. They are built based on known data. In the first step, the data are bootstrapped, which means that random instances of the original data set set are taken with replacement. With this bootstrapped data, decision trees with randomly selected attributes of the nodes, randomly selected number of nodes, and random order of nodes, are created. The advantage of this multitude of trees (this could well be 100 trees) is the large variety, which makes this approach very effective for classification. After this “training process,” an unknown instance can be classified. Each tree gives a vote to classify a new object based on the corresponding attributes. The final classification of the “forest” follows the label with the most votes.

#### 3.1.2 Clinical application

An exciting field of application is the prediction of disease outcomes based on predefined parameters. For example, this model has been successfully applied to predict in‐hospital mortality in heart failure patients.^7^

### 3.2 K‐means cluster

#### 3.2.1 Intuition

**Figure 3:**
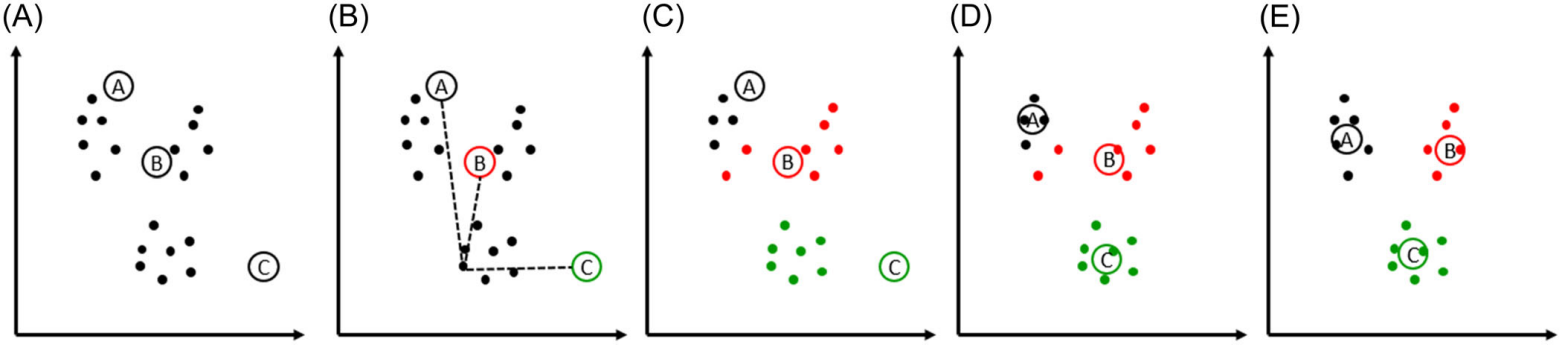
K‐means cluster. The algorithm k‐means cluster can be divided into six steps: (1) The number of clusters is defined (in the present: 3). (2) The cluster centers are randomly selected: black, red, and green (A). (3) The Euclidean distance is calculated for each point to the three cluster centers. Shown is an example of one point (B). (4) Each point is assigned to the next cluster according to the lowest Euclidean distance (C). (5) Next, the mean of each cluster is calculated, and the “new” center is located there (D). (6) Steps 3–5 are repeated until no change in the cluster centers and the optimal center locations are found (E).

K‐means cluster is a form of data analysis in which data is assigned to clusters. The number of clusters is *k*, which gives this method its name. The optimal cluster centers are found iteratively. The best number of clusters can be calculated by the elbow method. For details of this algorithm, see Figure 3. This principle seems simple at first glance, but the algorithm also provides excellent results in higher dimensional datasets with many instances.

#### 3.2.2 Clinical applications

K‐means cluster is essential in many clinical settings, where different entities are subsumed under umbrella terms. In this context, cluster analysis identifies disease subgroups with different pathogenesis and clinical outcomes. For example, the cluster identification technique has been used to identify specific phenotypes in pediatric patients with dilated cardiomyopathy associated with a bad outcome.^8^ Another example is the automated detection of coronary artery disease subgroups using phenotypic and genetic variables of 1329 patients.^9^

### 3.3 Convolutional neural networks (CNN)

#### 3.3.1 Intuition

A unique and more sophisticated kind of supervised learning are CNNs. They are described here in more detail as they are powerful and widely distributed. The development of CNNs shows how brain architecture is adapted for computational science.

In the 1960, the two Nobel Price winners, Torsten N. Wiesel, and David H. Hubel, explored the function of the visual cortex in the optical cortex of cats. The neuroscientist recognized that certain cells in the optical cortex fire according to the orientation of objects. This means, for example, that some cells fire at horizontal lines and others at vertical lines. Besides these cells recognizing only very basal orientations, they also postulated cells and networks that recognize complex patterns.^10^

**Figure 4:**
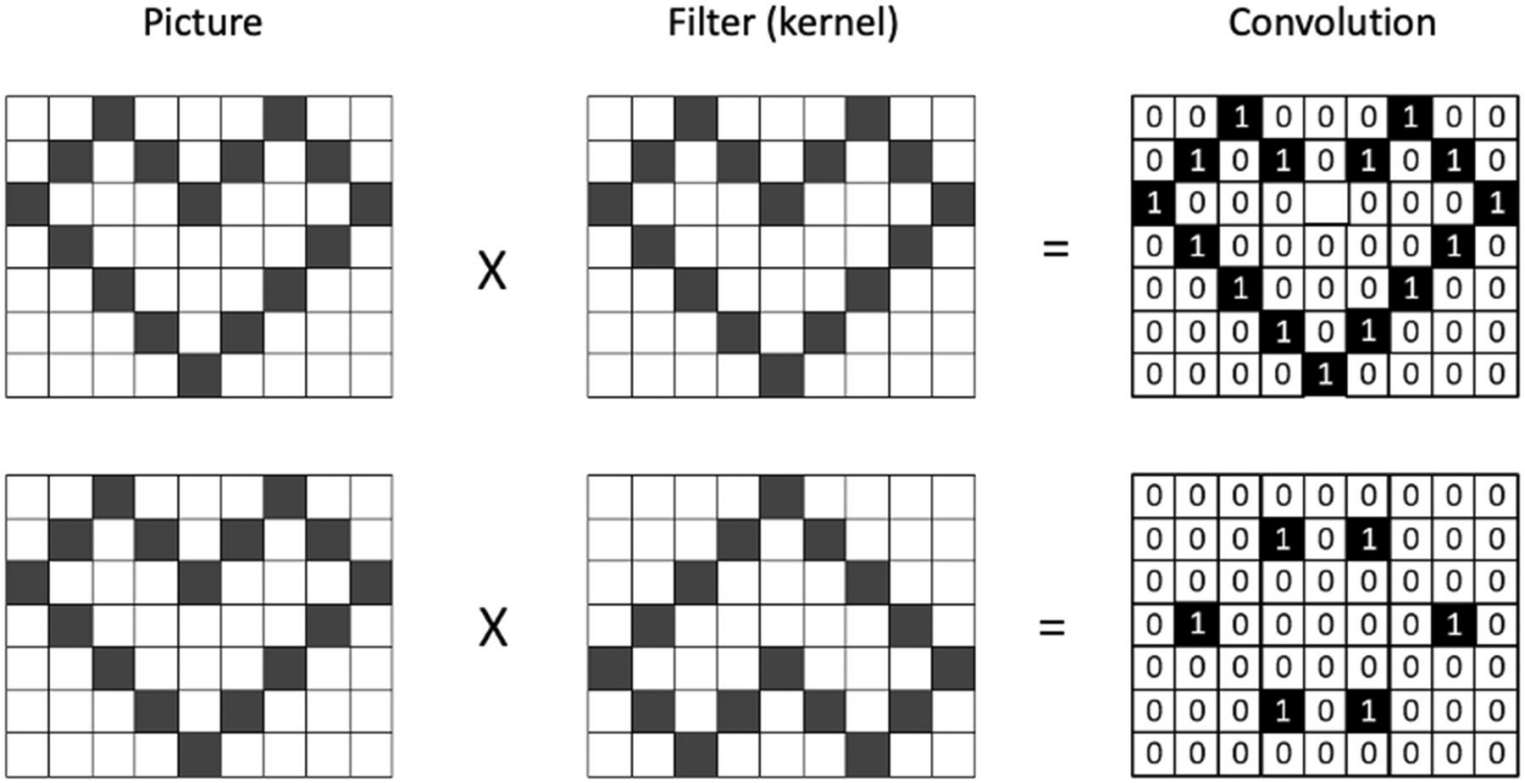
Convolutional neural network (CNN). The basic idea of a CNN is to evaluate the match between a given picture and a predefined filter. In this concept, the filter is small in comparison with the size of the picture. Therefore the filter slides over the image. The figure shows an image of a heart. The upper filter also shows a heart with a 100% match. The filter in the lower panel is an inverted heart with a poor match. The convolution can calculate the measure of agreement. A numerical agreement value can be obtained by multiplying the pixel values of the picture with the filter values. However, other mathematical operations can also be applied. In the present example, a high value stands for a high match.^12^

A CNN operates similarly. Convolution is a mathematical operation that creates a third function on two other functions. Although CNNs have many different applications, I would like to explain the principle using the example of image recognition. A given image is to be classified. To achieve this goal, the image is compared with different templates, also called filters or kernels, in computer science. These templates are usually predefined patterns. The CNN evaluates how high the match is.^11^ Since a black‐and‐white image is an array of pixels encoding the intensity of the grayscale, mathematical operations values can be subjected to these pixels. Using different filters, simple and more complex features can be identified in images. To achieve this goal, not a big filter is placed over the whole picture, but a small filter slides in lines over the big picture. In this context, the filters can also be viewed as small images. To put it very vividly, the algorithm looks at whether these tiny images of only a few pixels (e.g., 8 × 8 pixels) can be found in the big picture. By sliding the filter over the big picture, we get at every position a number presenting the degree of match. You get a filtered picture if you line up these numbers at the corresponding positions. Using several different filters, one image becomes a stack of filtered images. After performing further operations like normalization (rectified linear unit—ReLU) and pooling, this filtered image stack is merged into a fully connected layer. From then on, the following processes occur as in a usual deep neural network (Figure 4).

#### 3.3.2 Clinical application

This type of network is particularly valuable in image classification. In many fields of medicine, images from different disease entities must be classified. In cardiology, CNNs effectively classify electrocardiograms to distinguish cavotricuspid isthmus dependence from other atrial tachycardia mechanisms.^13^ Another clinically important application in cardiology is the classification of functionally significant coronary stenosis in coronary CT angiography.^14^ Although CNNs were initially used in image recognition, they are also a powerful tool in other fields. This is intuitively logical since images are only typical data arrays. For example, reliable classification of heart sounds can be achieved after processing audio sequences into data resembling image data.^15^

### 3.4 Recurrent neural networks (RNN)

#### 3.4.1 Intuition

Until now, we have only discussed feedforward neural networks, meaning that information flows only in one direction. Another subtype of supervised learning is RNNs. In contrast to feedforward networks, they have a backward‐directed loop. Layers are connected to the next layer, which then connects back to the former layer forming a loop. By this mechanism, sequential data can be processed for prediction. Taking the timeline as an example of sequential events, statements about the future are made based on past and current findings. Because of this mode of operation, RNNs most closely resemble short‐term memory of the frontal lobe in humans. When a ball is thrown, a human can only make a meaningful statement about the next position if the former positions are considered. RNNs make similar sequential predictions.

#### 3.4.2 Clinical applications

Due to this architecture, they are especially suitable for analyzing sequential events like electrocardiograms,^16^ audio data in auscultation,^17^ and language processing.^18^ Other promising fields are the prediction of in‐hospital cardiac arrest or acute kidney injury in hospitalized patients. The chronological sequence of laboratory values and diagnostic results are analyzed in these cases.^19^, ^20^

### 3.5 Self‐supervised representation learning

#### 3.5.1 Intuition

Supervised learning is dependent on labeling a large amount of data. However, data labeling is expensive; thus, high‐quality labeled data sets are limited. Therefore, new concepts have been developed in recent years. Self‐supervised learning is an algorithmic approach mimicking how children learn in the first 24 months. During this period, children primarily learn by observing. While observing, the brain learns to predict the future based on the past or predict the whole picture by showing only parts of it. The prediction is then compared with reality, and the neural network in the brain is continuously refined. A concept of the world is created through observation.

Putting this in algorithms means that the learning process is divided into two steps: (1) A proxy task is designed (e.g., a picture is rotated or divided into parts), and the model is trained on this pretext task. During this process, the model learns the representational features of the image.^21^ (2) The model is trained with very few labeled data during the downstream task.

#### 3.5.2 Clinical application

This approach has been successfully applied in medical image classification.^22^, ^23^ In cardiology, self‐supervised learning has been applied for correcting coronary MR angiography reconstruction for respiratory artifacts.^24^

### 3.6 Reinforcement learning

#### 3.6.1 Intuition

Reinforcement learning uses the concept of operant conditioning introduced by Skinner in behavioral science. An agent learns what action to take based on predefined rules to get later the highest reward. The agent iteratively makes different decisions until it finds the optimal policy to achieve this goal. In other words, the agent learns through trial and error. This concept can be traced back to the neural level, as dopaminergic neurons show activity fluctuations depending on success or failure.^25^

**Figure 5:**
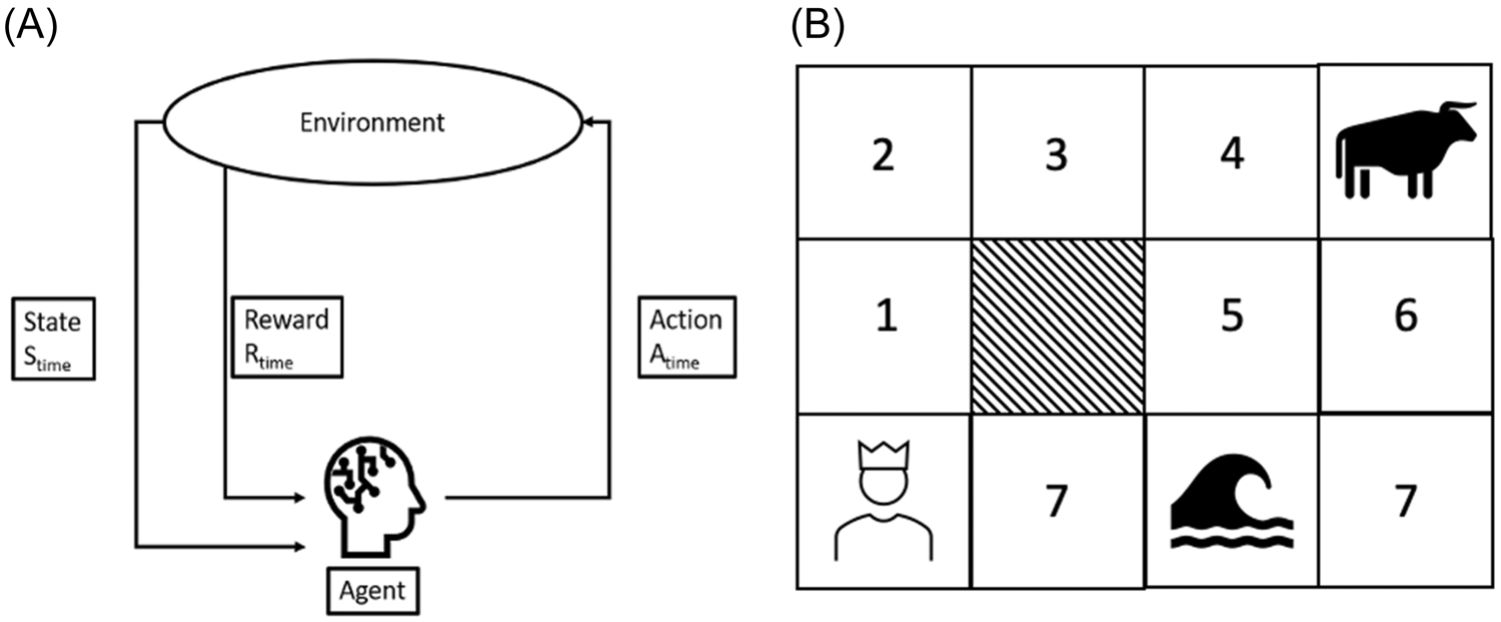
Reinforcement Learning. (A) Scheme of the basic concept of reinforcement learning. The agent acts in the environment and reaches new states and rewards. This leads to a feedback loop. On the right side (B), you see a practical example. The prince must find the Minotaur. The maze has three predefined fields: an insurmountable streamlined obstacle, a lake, and the Minotaur. Now, the prince starts moving. If he chooses the way up, he has the chance to find the Minotaur, which ultimately leads to a reward of 1. If he chooses the right way, he will drown in the lake, leading to a reward of −1. When the prince takes the first step, he can only explore the environment because he knows nothing about it. If he goes up to field 1, the value of his state increases as he is closer to the Minotaur, although he gets no immediate reward. If he goes right to field 7, the value of his state decreases as he is near the lake, which is associated with a negative reward. Following this concept, each field in the maze can be assigned a value. These values can be calculated with the Bellman‐Equation.

The essential components of a reinforcement framework are an agent and an environment. The agent interacts with the environment. To bring a mythological example: The prince Theseus goes through a maze looking for the Minotaur (Figure 5). In this case, Theseus is the agent, and the labyrinth is the environment. The agent takes action in the environment and gets a reward for it. The full reward is usually not immediate, as many steps must be taken. After each step, the agent is in a new state. This new state has a specific value. To return to the maze example, the value of a state is higher the closer the agent is to the Minotaur.

But how does the agent know what step to take at the start? Principally, the agent can do two kinds of actions. It can exploit the environment by taking steps with known favorable rewards. But the agent can also explore the environment, which means that the agent does a random step to determine whether this new move goes along with more or less reward. It is the mixture of exploration and exploitation that makes the learning of the algorithm. Richard Bellman put this in a mathematical form in the 50th of the last century.^26^

#### 3.6.2 Clinical applications

Reinforcement learning can be used in medical situations where sequential decision‐making is mandatory. Its area of operation can therefore be clinical‐decision support systems. In this context, a clear reward function must be defined. First experiences have been made with these algorithms concerning mechanical ventilation or sedation dosing in intensive care medicine.^27^, ^28^ Another interesting approach in cardiology is using a reinforcement model for dose‐finding the antiarrhythmic drug dofetilide.^29^

### 3.7 Transformers

#### 3.7.1 Intuition

Actually, transformer models are beyond the scope of this review. However, since a whole new level of AI has been reached with them, they will be briefly discussed at this point. They belong to the generative AI models that generate new data based on existing data. These new data can be text, images, or code. Giving an input, also called “prompt,” to the generative model gives back the statistically most probable answer. In this characteristic, they differ from the former discriminative models. Discriminative models give as output the probability of belonging to a label.

The history of transformers started with the paper “Attention is all you need,” which is also the core concept of this new architecture.^30^ The attention mechanism tries to identify the meaning of each piece of information in a sequence of information. In this ability, the algorithm resembles human intelligence. For example, when a human sees a picture, the human brain can distinguish important from unimportant parts. The brain then directs attention to these crucial parts and extracts features from these regions.

Transformers belong to semi‐supervised learning algorithms. They are pre‐trained by unlabeled datasets and fine‐tuned by supervised learning. In contrast to RNN, transformers analyze data not sequentially but on the attention algorithm. That allows transformers to run parallel analyses, accelerating the learning process. The LLMs like GPT‐3 and ‐4 (generative pretrained transformer 3rd and 4th generation) BERT (Bidirectional Encoder Representations from Transformers), and Claude are the well‐known transformers.

#### 3.7.2 Clinical application

One of the most groundbreaking successes of transformers in medicine is the prediction of the three‐dimensional structure of proteins based on the sequence of proteins using the AlphaFold algorithm.^31^, ^32^ So far, there have been only isolated applications of transformers in cardiology. Recently it has been reported that Chat‐GPT can give reasonably correct recommendations for cardiovascular disease prevention.^33^

### 3.8 Graph neural networks (GNN)

#### 3.8.1 Intuition

Classical ML has the problem of recognizing contextual relationships. However, that is a strength of GNNs. They can be applied in both supervised and unsupervised learning settings. A graph is a structured way to represent data about the relationship between parameters. In principle, a graph consists of nodes, also called vortices, and edges, also called links. A typical example of a graph is a social network where the nodes are the persons, and the edges are the relationships the persons have with each other. The nodes could contain personal characteristics like hobbies, attended school, etc. The edges are the relationships between the nodes. Using this basal architecture, triples can be created between two nodes, like “the author is born in Germany.” In this case, the nodes are “the author” and “Germany,” and the edge is “is born.” Another example is molecules, where atoms are the nodes and the bonds are the edges. After establishing the nodes and edges, an adjacency matrix representing the connections between the nodes, and a degree matrix, representing the number of connections of each node can be created.

#### 3.8.2 Clinical applications

GNN has been used in cardiology to classify polar maps in cardiac perfusion imaging.^34^ Another field is the estimation of left ventricular ejection fraction in echocardiography.^35^

## 4 LIMITATIONS OF AI

Although AI has achieved groundbreaking success in many fields of medicine, it is far from perfect. There are three significant limitations:

a. Data bias and algorithmic fairness.
  In supervised learning, prediction quality essentially relies on the quality of the data on which the algorithm has been trained. In this scenario, the classification of the ground truth is crucial. It is plausible that an image classifieronly works well when pictures of the training data are labeled accurately. In the same category falls data bias. An X‐ray classification algorithm trained with data from men cannot be applied to women. However, data bias is often not as obvious but can be very subtle. As data are not static, data shifts can cause significant problems. This category also includes algorithmic fairness. As algorithms are often trained with historical data, this can lead to discrimination of vulnerable groups. A prominent example is an algorithm used to manage the health of millions of patients has been published. This algorithm incorrectly indicated that black patients are considerably sicker than white patients.^36^
b. Security risks.
  Adversarial attacks are malicious inputs to ML algorithms that intentionally try to cause misclassification. Although no examples have been reported, the threat is considered real, and appropriate countermeasures are currently being developed.^37^
c. Impact on clinical outcome.

Although more and more studies with AI algorithms are prospective, the gold standard in medicine remains the randomized clinical trial (RCT) evaluating the effect of an algorithm on the clinical outcome in patients.^38^ In concrete terms, it is insufficient to be as good as the ground truth, however defined. Ultimately, the clinical outcome in RCTs must be equal to or better than the outcome achieved by human intelligence.

## 5 PERSPECTIVE

In many fields of AI, attempts have been made to emulate the structures of the human brain. However, the performance and capabilities of human intelligence are unattainable with current algorithms. Computational neuroscience is a research area between information technology and neurosciences, which brings scientific activities in these fields together. Although there are many similarities between ANNs and the human brain, there are also essential differences.

AI represents a rapidly developing field. Its use can improve diagnosis and therapy in many areas of medicine. However, it becomes essential that we use AI wisely and that we know how it works. It must not be a black box for doctors. Even though algorithms can operate autonomously in many cases, it is still essential that physicians understand them. The ultimate decision on diagnosis or therapy must remain with the physician. This overview should help to get a basic understanding of the fundamental algorithms of AI. However, this review can only present a small selection of algorithms and therefore does not claim to be complete.

## Supporting information

Supporting information

## Data Availability

Data sharing is not applicable to this article as no datasets were generated or analyzed during the current study.
