# Supplementary material for: Principles of artificial intelligence and its application in cardiovascular medicine
Source: Clin Cardiol. 2023 Sep 18;47(1):e24148. doi: 10.1002/clc.24148 (PMC10766001; doi:10.1002/clc.24148)
Supplement: Supplementary file 1 — Supporting information [file CLC-47-e24148-s001.docx]

Table 1.

Type of problem Algorithm Aim of the study

Outcome prediction random forest Race-specific and race-agnostic prediction of in-hospital

Segar et al.^6^ mortality of heart failure patients

Subgroup identification K-means cluster Subgroup detection in patients with coronary artery

Flores et al.^8^ disease to apply tailored therapy

Examination prediction CNN Detection of aortic stenosis/classification of

Voigt et al.^13^ heart sounds by using audio files

Outcome prediction recurrent neural Prediction of in-hospital cardiac arrest

Kwon et al.^17^ network (RNN)

Decision-making reinforcement learning Dosing adjustment for the antiarrhythmic drug dofetilide

Levy et al.^27^

Image segmentation self-supervised learning Short-axis image and long-axis image segmentation

Bai et al.38 in cardiac MR scans

Parameter prediction graph neural Prediction of ejection fraction in echocardiography

Mokhtari et al.^33^ network

The table shows an overview of the most important algorithms, examples of their application in cardiology, and the specific

problem they are designed to solve.
